# Associations between Cooking at Home and Nutrient and Food Group Intake among Female University Students: A Cross-Sectional Analysis on Living Arrangements

**DOI:** 10.3390/nu15041029

**Published:** 2023-02-18

**Authors:** Hana Hamade, Aoi Moriyasu, Osamu Kushida

**Affiliations:** 1 Department of Nutrition, Faculty of Health Science, Kio University, Nara 635-0832, Japan; 2 Department of Nutrition and Life Sciences, School of Food and Nutritional Sciences, University of Shizuoka, Shizuoka 422-8526, Japan

**Keywords:** cooking frequency, diet quality, university students, living with families, living alone

## Abstract

This cross-sectional study examined the association between cooking frequency and nutrient and food group intake among female university students with different living arrangements in Japan. Nutrient and food group intakes were assessed using a validated, brief, self-administered diet history questionnaire. Cooking frequency was measured using a single question on a five-point scale. The questionnaire also asked about living arrangements. Of the 91 respondents, 75 females were analyzed. Regarding cooking frequency, cooking at least 1–2 days a week was classified as cooking, and the “cooking yet living with families” group was compared with the “not cooking and living with families” and “cooking and living alone” groups. Based on the intakes of the “cooking yet living with families” group, the “not cooking and living with families” group consumed more total fat (29.5% energy vs. 33.0% energy, *p* = 0.010) and fewer cereals (224.8 g/1000 kcal vs. 179.6 g/1000 kcal, *p* = 0.007), and the “cooking and living alone” group consumed more confectionaries (21.0 g/1000 kcal vs. 34.5 g/1000 kcal, *p* = 0.023). This study showed that female university students who cook at least once a week and live with their families may have better diet quality than those who do not cook and live with their families and those who cook and live alone.

## 1. Introduction

A healthy diet is important for the prevention of noncommunicable diseases. A recent large cohort study suggested that a balanced intake of cereals, vegetables, protein foods, fruits, dairy products, and confectionaries contributes to longevity by reducing the risk of death, primarily from cardiovascular disease, in the Japanese population [1]. Unhealthy eating behavior is especially common among adolescents and young adults. A previous literature review indicated that university students in several countries have unhealthy eating behaviors, such as a lower intake of fruits and vegetables, than is internationally recommended [2]. Younger adults in Japan are also characterized by a higher consumption of meat and a lower consumption of fish and vegetables [3]. Regarding dietary patterns, higher factor scores for high-meat and low-fish patterns and lower factor scores for vegetable patterns were associated with lower intake levels of beneficial nutrients [3]. Growth and development during adolescence may significantly impact the health of the individual in later life, as well as their potential children [4]. Therefore, it is important to identify the factors associated with eating behavior changes in adolescents and young adults.

Previous systematic reviews have examined the association of cooking and food preparation with diet and health [5,6]. For example, a study by Laska et al. showed that emerging adult food preparation predicted better dietary quality five years later [7]. Several other studies showed that cooking at home was associated with a healthy diet [8,9] and survival [10]. Another study reported an association between less home cooking by caregivers and higher diastolic blood pressure and lower HDL cholesterol [11] in children.

Recent studies focusing on cooking among adolescents and students have found that the frequency of cooking is associated with greater fruit and vegetable intake [12,13,14]. However, because these studies did not consider differences between individuals living with their families or those living alone, the association between cooking frequency and the intake of nutrients and food groups by living arrangement is not clear. Almost all Japanese students live with their families or alone [15,16]. Furthermore, among Japanese students who live with their families, most of the food is cooked by their parents [17]; thus, the cooking frequency of students may vary with living arrangements. Students who live with their families yet cook might have better diet quality.

Since young adults in Japan have unhealthy eating habits and most of them live with their families or live alone, it is important to examine eating-related behaviors in the context of living arrangements. Therefore, this study aimed to examine the association between cooking frequency and the intake of nutrients and food groups based on the living arrangements of Japanese university students.

## 2. Materials and Methods

### 2.1. Participants

A cross-sectional study consisting of a self-administered questionnaire was conducted in September 2016. The participants were the entire population of university students from a health support student organization at Kio University (*n* = 151). Kio University is a private university with approximately 2000 students in Nara Prefecture, Japan. The student organization includes undergraduate students from the departments of Physical Therapy, Nursing, Nutrition, Education, and Environmental Design. Questionnaires were distributed to each participant on campus in mid-September via a departmental representative from each academic year. Participants were asked to return the questionnaires at an organization activity at the end of the same month or drop them off at the authors’ laboratories. Of the 151 participants, 11 were excluded because they were unavailable mainly due to off-campus training. Questionnaires were distributed to 140 participants, and 91 responses were obtained (for a response rate of 65.0%). The respondents were identified by their names on the return envelopes, and individuals who omitted responses or provided inconsistent responses were surveyed again.

This study was conducted after review and approval by the Research Ethics Committee of Kio University (Approval No. H28-32).

### 2.2. Measures

The participants’ habitual intakes of nutrients and food groups were assessed with a brief-type self-administered diet history questionnaire (BDHQ), which was validated using a semi-weighed 16-day dietary record in Japan [18,19]. The BDHQ is a non-quantitative food frequency questionnaire (FFQ) assessing dietary habits during the preceding month, consisting of the intake frequency of 46 food items, the daily intake of rice and miso soup, the frequency of drinking and amount per drink for alcoholic beverages, and diet histories (e.g., assessment of cooking methods). In the validation study of the FFQ, dietary records are likely to have the least correlated errors [20]. The correlation coefficients of nutrients and food group intakes between the BDHQ and dietary records were calculated using the density method as a percentage of energy or as intake per 10 MJ [18,19]. Although participants ranged in age from 31 to 69, the median (interquartile range) Pearson correlation coefficients for nutrients were 0.49 (0.38 to 0.57) for women and 0.49 (0.36 to 0.59) for men [18], and the median (range) Spearman’s correlation coefficients for food groups were 0.44 (0.14 to 0.82) for women and 0.48 (0.21 to 0.83) for men [19]. Of the 42 nutrients calculated from the BDHQ, 20 nutrients were evaluated in this study. The 20 nutrients were those for which the estimated average requirement (EAR) or tentative dietary goal for preventing lifestyle-related diseases (DG) are specified by the Dietary Reference Intakes for Japanese (2015) [21]. Of the 18 food groups classified in the Standard Tables of Food Composition in Japan 2015 (Seventh Revised Edition) [22], 15 food groups were also evaluated. Incidentally, vegetables were calculated by dividing them into green and yellow vegetables and other vegetables, and mushrooms and algae were classified as other vegetables. Nuts and seeds, and prepared food groups listed in the Standard Tables of Food Composition in Japan 2015 (Seventh Revised Edition) could not be evaluated in this study because they were not calculated by the BDHQ. Specific food items and food group classifications are summarized by Kobayashi et al. [19].

The cooking frequency question was a single item, as in previous studies [8,9,10,11,12,13]. Specifically, the participants were asked, “How often do you cook?” and their answers were measured on a 5-point scale: not at all, 1–2 days a week, 3–4 days a week, 5–6 days a week, and every day. To measure whether the participants cooked at all, the authors provided a definition of cooking. A definition of cooking included tearing food (e.g., lettuce) by hand but excluded boiling water or simply heating meals. Because several previous studies have shown that cooking frequency is associated with cooking skills [23,24,25,26], the validated cooking skills scale [26] with Japanese female students was used to examine the validity of the cooking frequency question. There were five cooking skill items: “I can cut ingredients appropriately for each dish”, “I can arrange the way I make a dish”, “I have a knack for making food taste good”, “I can think of steps to make meals more efficiently”, and “I can prepare good meals without spending money”. A total score of 0–5 was obtained by summing the answers of cannot (0) and can (1).

The participants were asked to report the following sociodemographic characteristics: sex, age, height, weight, department of study, and living arrangement. Body mass index (BMI) was calculated by dividing the weight (kg) by the square of the height (m). Living arrangements were obtained by “family home” or “live alone”. Those who lived with their families were asked who is in charge of the cooking, and those who were not in charge of the cooking were also asked how often the cook is absent. As an additional characteristic, meal-making motivation, which may be associated with whether the participant cooks, was also measured. The meal-making motivation was measured by a validated scale in Japan, which consists of two subscales: “spontaneous motivation” and “motivation corresponding with expectations” [27]. The spontaneous motivation subscale consists of two items: “because I enjoy making meals” and “because I like making meals”. The other subscale, motivation corresponding with expectations, consists of three items: “because there are people who look forward to meals”, “because people who eat meals express their gratitude”, and “because people request that I make meals for them”. Responses were obtained on a 6-point scale ranging from not at all applicable (0) to very applicable (5) for each of the items of the two subscales. The range of the total score was 0–10 for “spontaneous motivation” and 0–15 for “motivation corresponding with expectations”.

### 2.3. Data Analysis

Differences in cooking frequency and living arrangements by groups will be defined in the following section. Differences in sociodemographic characteristics as well as in charge of cooking, frequency of absence of cooks, meal-making motivation, and cooking skills among the groups were examined by the chi-square test for sociodemographic characteristics on categorical variables, and the Mann–Whitney U test for meal-making motivation and cooking skills on quantitative variables with Bonferroni correction. Differences in the intake of nutrients and food groups were examined using Dunnett’s *t* test with the group as the independent variable. Histograms and normal probability plots were used to confirm the normal distribution of nutrients and food groups and an analysis using log-transformed values for vitamin A intake. The level of significance was set at *p* < 0.05, and Bonferroni corrections were applied as appropriate. IBM SPSS Statistics 27 (IBM Japan, Ltd., Tokyo, Japan) was used for all statistical analyses.

## 3. Results

Of the 91 respondents, 75 were included in the analysis, excluding 12 males (13.2%) because the focus of the study is females, and four individuals who reported in the BDHQ that their energy intake was less than half the energy requirement for the lowest physical activity category according to the recommended Dietary Reference Intakes for Japanese [17]. Cooking frequency varied greatly with living arrangements (Table 1). Only 6% of those who lived with their families answered that they cooked 3–4 days a week or more, and 0% of those who lived alone answered that they did not cook at all. Therefore, the respondents who answered at least 1–2 days a week were classified as cooking, not at all were classified as not cooking, and the “cooking yet living with families” group was compared with the “not cooking and living with families” and the “cooking and living alone” groups. When respondents who lived with their families were asked about the person in charge of cooking, 90.6% of them answered their parents. The median (25–75 percentile) score for cooking skills was 2 (1–4) overall. Spearman’s correlation coefficients between the score of cooking skills and cooking frequency measured on the 5-point scale confirmed a good correlation (ρ = 0.45, *p* < 0.001).

The sociodemographic characteristics, in charge of cooking, frequency of absence of cooks, meal-making motivations, and cooking skills of the participants are shown in Table 2. The mean age of the participants was 19.4 years. There were no statistically significant differences in age, BMI, in charge of cooking, and frequency of absence of cooks between the groups. Regarding departments, 13 (39.3%) of the respondents in the “cooking yet living with families” group studied in the Department of Nutrition, compared to 13 respondents (41.9%) in the “not cooking and living with family” group who studied in the Department of Physical Therapy, indicating a significant difference between the groups (*p* = 0.024). For meal-making motivation, the median (25–75 percentile) score for spontaneous motivation in the “not cooking and living with families” group (2 (0–6)) was lower than in the “cooking yet living with families” group (8 (6.5–10), *p* < 0.001). The median (25–75 percentile) score for motivation corresponding with expectations also showed a low value in the “not cooking and living with families” group (2 (0–8)), compared to that of the “cooking yet living with families” group (10 (6.5–12.5), *p* < 0.001).

Regarding cooking skills, a comparison between groups showed a significant difference between those in the “not cooking and living with families” group, 1 (0–2), and those in the “cooking yet living with families” group, 2 (1–4) (*p* = 0.009). On the other hand, those in the “cooking and living alone” group, who cooked more frequently, had a significantly higher score of 4 (3–5) (*p* = 0.024).

The nutrient and food group intakes of the participants are shown in Table 3 and Table 4. Among the nutrients, total fat intake was significantly higher in the “not cooking and living with families” group, at 33.0 ± 4.8% energy, versus 29.5 ± 4.8% energy in the “cooking yet living with families” group (*p* = 0.010). Zinc intake was significantly lower in the “cooking and living alone” group, at 4.1 ± 0.5 mg/1000 kcal, versus 4.7 ± 0.5 mg/1000 kcal in the “cooking yet living with families” group (*p* = 0.007). Copper intake was significantly lower in the “not cooking and living with families” group, at 0.55 ± 0.08 mg/1000 kcal, versus 0.60 ± 0.08 mg/1000 kcal in the “cooking yet living with families” group (*p* = 0.028). Of the food groups, cereal intake was significantly lower in the “not cooking and living with families” group, at 179.6 ± 59.7 g/1000 kcal, versus 224.9 ± 50.2 g/1000 kcal in the “cooking yet living with families” group (*p* = 0.007). Fat and oil intakes were significantly higher in the “not cooking and living with families” group, at 7.9 ± 2.8 g/1000 kcal, versus 6.5 ± 2.3 g/1000 kcal in the “cooking yet living with families” group (*p* = 0.041). Confectionary intake was significantly higher in the “cooking and living alone” group, at 34.5 ± 18.5 g/1000 kcal, versus 21.0 ± 14.4 g/1000 kcal in the “cooking yet living with families” group (*p* = 0.023). Seasoning and spice intakes were significantly higher in the “cooking and living alone” group, at 135.2 ± 63.2 g/1000 kcal, versus 92.6 ± 50.5 g/1000 kcal in the “cooking yet living with families” group (*p* = 0.021).

## 4. Discussion

This study examined the association of cooking frequency with the intake of nutrients and food groups among female university students with different living arrangements. Because those who lived with their families cooked less frequently, this study was designed to compare those who cooked and those who did not cook. As a result, the “cooking yet living with families” group consumed a lower proportion of total fat and consumed more cereals than the “not cooking and living with families” group, and fewer confectionaries than the “cooking and living alone” group. This result was similar to a previous study showing that those who cooked dinner more frequently consumed less fat and sugar [6]. Although the tentative dietary goal for total fat intake is set at 20 to 30% energy in Japan [21], those for whom total fat constitutes 30% or more energy are most often young adults [28], with half of the women in their 20s falling into this intake category [29]. While a large cohort study has shown that diets in which carbohydrates constitute 50–55% energy have the lowest risk of mortality [30], those who did not cook and lived with their families tended to have a slightly lower average carbohydrate to energy ratio of 50.1% and significantly lower cereal intake. Furthermore, although the participants were of different ages, a high intake of confectionaries is associated with poor sleep quality in middle-aged women [31]. These results suggest that cooking is associated with better diet quality, even if the university students who live with their families are not in charge of cooking. Although previous studies suggested that having the habit of cooking at home may lead to improved diet and health [7,8,9,10,11], the results of this study suggest that it may also be important for students to have cooking experience while living with families.

One of the reasons cooking is preferable, even among those who are not in charge of cooking, is the family connection. In a study of the parents of toddlers and preschoolers, meals cooked by parents and children together were associated with the intake of healthy diets, including fish, soybeans/soy products, and vegetables for children [32]. In another study of elementary school children, children’s involvement in meal preparation was found to be associated with their cooking skills [33]. Therefore, cooking with those in charge of cooking may lead to the adoption of more healthful diets for university students by improving their cooking skills and the quality of their family meals.

The “cooking yet living with families” group had higher meal-making motivation, especially spontaneous motivation, than the other two groups. A lower spontaneous motivation may be a mediating factor as to why those who live alone had more confectionaries than those who live together and cook, even though they cook. Furthermore, to increase the cooking frequency for individuals living with their families, it may be beneficial to encourage their spontaneity, such as to enjoy and like making meals.

Limitations of this study include, first, that the small sample size did not allow the consideration of cooking frequency or adjustment for potential confounders. Because the response rate was not very high (65%), as well as the participants being health support students, they may participate in cooking behaviors more than the average population. This study was also unable to examine men, who generally cook less frequently. In this study, although the “cooking yet living with families” group tended to have higher overall nutrient intake, many nutrients were not significantly different. Because this study included the entire population of one student organization, the authors did not perform a power analysis to determine the sample size. Further studies from a variety of areas with larger samples that can be divided by cooking frequency are needed. Second, because this is a cross-sectional study, the causal associations between cooking frequency and intake of nutrients and food groups have not been established. Third, all the variables measured in this study were self-reported. Although cooking frequency, meal-making motivation, and intake of nutrients and food groups by BDHQ have all been tested for validity, future studies using scales that have been tested for reliability as well as validity will be needed. Furthermore, although the cooking question was provided with a definition, the same response category could have varied in the degree of cooking. Despite these limitations, the strength of this study is that it not only examined the validity of cooking frequency but also considered variations in cooking frequency by living arrangements.

## 5. Conclusions

This study, which examined the association of cooking frequency with the intake of nutrients and food groups in students with different living arrangements, suggested that female university students who cook at least once a week and live with their families may have better diet quality than those who do not cook and live with their families and those who cook and live alone.

## Figures and Tables

**Table 1:** Cooking frequency among female university students by living arrangements (*n* = 75).

	Family Home (*n* = 64)	Live Alone (*n* = 11)
Cooking frequency		
Not at all	31 (48.4)	0 (0.0)
1–2 days a week	29 (45.3)	1 (9.1)
3–4 days a week	2 (3.1)	7 (63.6)
5–6 days a week	0 (0.0)	2 (18.2)
Every day	2 (3.1)	1 (9.1)

*n* (%).

**Table 2:** Association of sociodemographic characteristics with cooking among female university students (*n* = 75).

	Cooking yet Living with Families (*n* = 33)	Not Cooking and Living with Families (*n* = 31)	*p*	Cooking and Living Alone (*n* = 11)	*p*
Age (year) *			0.397		0.153
18 y	7 (21.2)	12 (38.7)		1 (9.1)	
19 y	14 (42.4)	10 (32.3)		2 (18.2)	
20 y	6 (18.2)	6 (19.4)		4 (36.4)	
21 y	4 (12.1)	3 (9.7)		3 (27.3)	
22 y	2 (6.1)	0 (0.0)		0 (0.0)	
25 y	0 (0.0)	0 (0.0)		1 (9.1)	
Body mass index (kg/m^2^) *			0.994		0.704
<18.5	3 (9.1)	3 (9.7)		1 (9.1)	
18.5≤, <25.0	28 (84.8)	26 (83.9)		10 (90.9)	
25.0≤	2 (6.1)	2 (6.5)		0 (0.0)	
Department *			0.024 ^‡^		0.542
Physical Therapy	8 (24.2)	13 (41.9)		4 (36.4)	
Nursing	7 (21.2)	11 (35.5)		2 (18.2)	
Nutrition	13 (39.4)	2 (6.5)		2 (18.2)	
Education	4 (12.1)	5 (16.1)		3 (27.3)	
Environment Design	1 (3.0)	0 (0.0)		0 (0.0)	
In charge of cooking *			0.112		
Themselves	1 (3.0)	0 (0.0)			
Parents	32 (97.0)	26 (83.9)			
Grandparents	0 (0.0)	3 (9.7)			
Other	0 (0.0)	0 (0.0)			
Missing data	0	1			
Frequency of absence of cooks *			0.109		
Often/Sometimes	16 (48.5)	9 (29.0)			
Not much/Almost never	16 (48.5)	21 (67.7)			
Meal-making motivation ^†^					
Spontaneous motivation	8 (6.5–10)	2 (0–6)	<0.001 ^‡^	6 (4–8)	0.044
Motivation corresponding with expectations	10 (6.5–12.5)	2 (0–8)	<0.001 ^‡^	9 (5–10)	0.215
Cooking Skill ^†^	2 (1–4)	1 (0–2)	0.009 ^‡^	4 (3–5)	0.024 ^‡^

*n* (%) or median (25–75 percentile). * Chi-square test. ^†^ Mann–Whitney U test. ^‡^ Significant difference with Bonferroni correction (*p* < 0.05/2).

**Table 3:** Association of cooking with nutrient intake among female university students (*n* = 75).

Nutrients	Cooking yet Living with Families (*n* = 33)	Not Cooking and Living with Families (*n* = 31)	*p* *	Cooking and Living Alone (*n* = 11)	*p* *
Total fat (% energy)	29.5 (4.8)	33.0 (4.8)	0.010	28.6 (4.8)	0.820
Saturated fat (% energy)	8.70 (1.77)	9.58 (1.70)	0.097	8.39 (1.97)	0.843
Protein (% energy)	16.0 (3.1)	15.2 (2.1)	0.365	14.4 (1.8)	0.140
Carbohydrate (% energy)	52.5 (6.3)	50.1 (6.3)	0.212	53.6 (5.3)	0.850
Total dietary fiber (g/1000 kcal)	5.6 (1.5)	5.8 (1.7)	0.862	6.1 (1.2)	0.614
Sodium chloride (g/1000 kcal)	5.4 (1.3)	5.4 (0.9)	0.980	5.6 (1.1)	0.782
Potassium (mg/1000 kcal)	1288 (310)	1333 (338)	0.812	1249 (314)	0.921
Vitamin A ^†^ (μg/1000 kcal)	330 (159)	396 (155)	0.118	347 (168)	0.960
Thiamine (mg/1000 kcal)	0.44 (0.08)	0.45 (0.09)	0.930	0.40 (0.06)	0.306
Riboflavin (mg/1000 kcal)	0.72 (0.15)	0.74 (0.14)	0.920	0.72 (0.18)	0.998
Niacin (mg/1000 kcal)	8.7 (2.5)	8.5 (1.8)	0.895	7.5 (1.7)	0.205
Vitamin B_6_ (mg/1000 kcal)	0.67 (0.16)	0.67 (0.16)	1.000	0.61 (0.12)	0.529
Vitamin B_12_ (μg/1000 kcal)	5.1 (3.4)	4.6 (1.8)	0.636	3.8 (1.4)	0.249
Folate (μg/1000 kcal)	158 (48)	168 (58)	0.649	167 (48)	0.855
Vitamin C (mg/1000 kcal)	54 (22)	60 (28)	0.477	60 (14)	0.696
Calcium (mg/1000 kcal)	306 (108)	298 (88)	0.932	292 (116)	0.898
Magnesium (mg/1000 kcal)	125 (28)	119 (24)	0.569	118 (22)	0.662
Iron (mg/1000 kcal)	4.0 (0.9)	3.9 (0.9)	0.977	3.9 (0.6)	0.950
Zinc (mg/1000 kcal)	4.7 (0.5)	4.5 (0.7)	0.302	4.1 (0.5)	0.007
Cooper (mg/1000 kcal)	0.60 (0.08)	0.55 (0.08)	0.028	0.56 (0.05)	0.382

Mean (standard deviation). * Dunnett’s *t* test. ^†^ Retinol equivalent (log-transformed value was used in the analysis).

**Table 4:** Association of cooking with food group intake among female university students (*n* = 75).

Food Groups (g/1000 kcal)	Cooking yet Living with Families (*n* = 33)	Not Cooking and Living with Families (*n* = 31)	*p* *	Cooking and Living Alone (*n* = 11)	*p* *
Cereals	224.9 (50.2)	179.6 (59.7)	0.007	218.1 (83.8)	0.931
Potatoes and starches	24.6 (20.3)	34.9 (22.7)	0.104	25.9 (18.8)	0.981
Sugars and sweeteners	2.7 (2.1)	2.8 (2.1)	0.982	1.7 (1.0)	0.286
Pulses	32.1 (20.3)	21.0 (20.7)	0.060	23.1 (18.4)	0.356
Dark green and yellow vegetables	55.7 (40.1)	59.5 (38.5)	0.902	49.7 (30.1)	0.869
Other vegetables ^†^	74.0 (34.2)	82.6 (37.9)	0.553	83.3 (38.5)	0.697
Fruits	51.3 (62.1)	56.6 (53.4)	0.905	58.3 (31.1)	0.914
Fish, mollusks, and crustaceans	43.3 (38.1)	33.4 (16.1)	0.292	32.6 (18.2)	0.463
Meat	50.1 (16.7)	53.6 (22.2)	0.703	38.0 (14.7)	0.131
Eggs	27.3 (13.0)	31.0 (14.4)	0.454	24.9 (10.7)	0.843
Milk and milk products	103.8 (60.6)	108.0 (64.1)	0.955	103.3 (75.1)	1.000
Fats and oils	6.5 (2.3)	7.9 (2.8)	0.041	6.6 (2.0)	0.988
Confectionaries	21.0 (14.4)	24.6 (14.4)	0.553	34.5 (18.5)	0.023
Beverages	229.4 (163.0)	260.1 (161.9)	0.683	268.0 (159.5)	0.732
Seasonings and spices	92.6 (50.5)	91.0 (34.5)	0.987	135.2 (63.2)	0.021

Mean (standard deviation). * Dunnett’s *t* test. ^†^ Includes mushrooms and algae.

## Data Availability

The datasets generated and analyzed during the current study are not publicly available due to privacy and ethical restrictions but are available from the corresponding author on reasonable request.

## References

[1] Kurotani K., Akter S., Kashino I., Goto A., Mizoue T., Noda M., Sasazuki S., Sawada N., Tsugane S., Japan Public Health Center Based Prospective Study Group (2016). Quality of diet and mortality among Japanese men and women: Japan Public Health Center based prospective study. BMJ.

[2] Bernardo G.L., Jomori M.M., Fernandes A.C., Proença R.P.C. (2017). Food intake of university students. Rev. Nutr..

[3] Okada E., Takahashi K., Takimoto H., Takabayashi S., Kishi T., Kobayashi T., Nakamura K., Ukawa S., Nakamura M., Sasaki S. (2018). Dietary patterns among Japanese adults: Findings from the National Health and Nutrition Survey, 2012. Asia Pac. J. Clin. Nutr..

[4] Norris S.A., Frongillo E.A., Black M.M., Dong Y., Fall C., Lampl M., Liese A.D., Naguib M., Prentice A., Rochat T. (2022). Nutrition in adolescent growth and development. Lancet.

[5] Mills S., White M., Brown H., Wrieden W., Kwasnicka D., Halligan J., Robalino S., Adams J. (2017). Health and social determinants and outcomes of home cooking: A systematic review of observational studies. Appetite.

[6] Reicks M., Kocher M., Reeder J. (2018). Impact of cooking and home food preparation interventions among adults: A systematic review (2011–2016). J. Nutr. Educ. Behav..

[7] Laska M.N., Larson N.I., Neumark-Sztainer D., Story M. (2012). Does involvement in food preparation track from adolescence to young adulthood and is it associated with better dietary quality? Findings from a 10-year longitudinal study. Public Health Nutr..

[8] Wolfson J.A., Bleich S.N. (2015). Is cooking at home associated with better diet quality or weight-loss intention?. Public Health Nutr..

[9] Wolfson J.A., Leung C.W., Richardson C.R. (2020). More frequent cooking at home is associated with higher Healthy Eating Index-2015 score. Public Health Nutr..

[10] Chen R.C.Y., Lee M.S., Chang Y.H., Wahlqvist M.L. (2012). Cooking frequency may enhance survival in Taiwanese elderly. Public Health Nutr..

[11] Tani Y., Fujiwara T., Isumi A., Doi S. (2020). Home cooking is related to potential reduction in cardiovascular disease risk among adolescents: Results from the A-CHILD study. Nutrients.

[12] Utter J., Denny S., Lucassen M., Dyson B. (2016). Adolescent cooking abilities and behaviors: Associations with nutrition and emotional well-being. J. Nutr. Educ. Behav..

[13] Hanson A.J., Kattelmann K.K., McCormack L.A., Zhou W., Brown O.N., Horacek T.M., Shelnutt K.P., Kidd T., Opoku-Acheampong A., Franzen-Castle L.D. (2019). Cooking and meal planning as predictors of fruit and vegetable intake and BMI in first-year college students. Int. J. Environ. Res. Public Health..

[14] Bernardo G.L., Rodrigues V.M., Bastos B.S., Uggioni P.L., Hauschild D.B., Fernandes A.C., Martinelli S.S., Cavalli S.B., Bray J., Hartwell H. (2021). Association of personal characteristics and cooking skills with vegetable consumption frequency among university students. Appetite.

[15] Goto M., Kiyohara K., Kawamura T. (2010). Lifestyle risk factors for overweight in Japanese male college students. Public Health Nutr..

[16] Kobayashi S., Asakura K., Suga H., Sasaki S., The Three-Generation Study of Women on Diets and Health Study Group (2017). Living status and frequency of eating out-of-home foods in relation to nutritional adequacy in 4,017 Japanese female dietetic students aged 18-20 years: A multicenter cross-sectional study. J. Epidemiol..

[17] Hori M., Hirasawa M., Isobe Y., Nagano H. (2012). Improvement in knowledge on cutting skills among junior college students after taking cooking classes. Bull. Gifu City Women’s Coll..

[18] Kobayashi S., Honda S., Murakami K., Sasaki S., Okubo H., Hirota N., Notsu A., Fukui M., Date C. (2012). Both comprehensive and brief self-administered diet history questionnaires satisfactorily rank nutrient intakes in Japanese adults. J. Epidemiol..

[19] Kobayashi S., Murakami K., Sasaki S., Okubo H., Hirota N., Notsu A., Fukui M., Date C. (2011). Comparison of relative validity of food group intakes estimated by comprehensive and brief-type self-administered diet history questionnaires against 16 d dietary records in Japanese adults. Public Health Nutr..

[20] Willet W.C., Lenart E.B., Willett W.C. (2013). Reproducibility and validity of food-frequency questionnaires. Nutritional Epidemiology.

[21] Ministry of Health, Labour and Welfare (2015). Dietary Reference Intakes for Japanese (2015). https://www.mhlw.go.jp/file/06-Seisakujouhou-10900000-Kenkoukyoku/Full_DRIs2015.pdf.

[22] Ministry of Education, Culture, Sports, Science and Technology (2015). Standard Tables of Food Composition in Japan -2015.

[23] Tani Y., Fujiwara T., Kondo K. (2020). Cooking skills related to potential benefits for dietary behaviors and weight status among older Japanese men and women: A cross-sectional study from the JAGES. Int. J. Behav. Nutr. Phys. Act..

[24] Soldavini J., Berner M. (2021). Characteristics associated with cooking frequency among college students. Int. J. Gastron. Food. Sci..

[25] Tani Y., Isumi A., Doi S., Fujiwara T. (2021). Associations of caregiver cooking skills with child dietary behaviors and weight status: Results from the A-CHILD study. Nutrients.

[26] Kamata H., Aikawa R. (2012). Attempts to develop “meal-making self-efficacy” scale. Res. Bull. Otsuma Women’s Univ. Home Econ..

[27] Kamata H., Ando S. (2014). Development of a meal-making motivation scale for homemakers. Jpn. J. Health Educ. Promot..

[28] Koyama T., Yoshita K., Okuda N., Saitoh S., Sakata K., Okayama A., Nakagawa H., Miyagawa N., Miura K., Chan Q. (2017). Overall nutrient and total fat intake among Japanese people: The INTERLIPID Study Japan. Asia Pac. J. Clin. Nutr..

[29] Ministry of Health, Labour and Welfare (2020). The National Health and Nutrition Survey in Japan, 2019.

[30] Seidelmann S.B., Claggett B., Cheng S., Henglin M., Shah A., Steffen L.M., Folsom A.R., Rimm E.B., Willett W.C., Solomon S.D. (2018). Dietary carbohydrate intake and mortality: A prospective cohort study and meta-analysis. Lancet Public Health.

[31] Katagiri R., Asakura K., Kobayashi S., Suga H., Sasaki S., The Three-Generation Study of Women on Diets and Health Study Group (2014). Low intake of vegetables, high intake of confectionary, and unhealthy eating habits are associated with poor sleep quality among middle-aged female Japanese workers. J. Occup. Health.

[32] Ishikawa M., Eto K., Miyoshi M., Yokoyama T., Haraikawa M., Yoshiike N. (2019). Parent-child cooking meal together may relate to parental concerns about the diets of their toddlers and preschoolers: A cross-sectional analysis in Japan. Nutr. J..

[33] Nozue M., Ishida H., Hazano S., Nakanishi A., Yamamoto T., Abe A., Nishi N., Yokoyama T., Murayama N. (2016). Associations between Japanese schoolchildren’s involvement in at-home meal preparation, their food intakes, and cooking skills. Nutr. Res. Pract..

